# Newspaper-Puffing of Surgical Operations, &c.

**Published:** 1858-04

**Authors:** 


### Newspaper-puffing of Surgical Operations, &c.

We have received a circular from Dr. E. S. Cooper, of San Francisco, California, intended to defend himself against the charge, upon the part of the Pacific Medical and Surgical Journal, of complicity in a newspaper puff of some surgical operation performed by him which had appeared in that city.

We have not been able, perhaps, to gather accurately all the points from the circular, but according to our understanding of the matter, *it is not a triumphant defence.*

In this connection, we would remark, that while, of course, no man should be held responsible for any notice of himself, which is published without his knowledge, it is not sufficient to free a medical man from the imputation of having himself complimented through the *newspapers*, to say, that he did not *solicit* it. Where an editor of a newspaper has been invited to attend an operation, or where special pains has been taken to inform him of some unusual skill in the profession, or, (as we have known,) of some *discovery* in medical science, we have no hesitation in saying, that the individual should be held responsible, and made to account at the bar of medical ethics, for a self-laudation, which is unprofessional, and grossly reprehensible. Non-professional persons, even though they be highly intelligent editors, cannot possibly be competent judges of medical matters—of what is *extraordinary* skill in surgery or medicine, or whether pretensions to *discoveries* in medical science are well founded or otherwise—and, besides, there is a recognized channel through which a medical man has an acknowledged right to communicate anything *to the world*, which he may suppose will contribute to science, or advance his repution. The Medical Journals of the country are open to all respectable medical men, through which they have the largest liberty to submit to their professional brethren whatever they may suppose to be original or valuable in the way of contributions to science; and he who is not willing to abide by the adjudication of those who it is manifest alone can be competent to judge of such matters, is unworthy of an honorable place in the ranks of a liberal and philanthropic profession.

